# Ultra-low-coverage genome-wide association study—insights into gestational age using 17,844 embryo samples with preimplantation genetic testing

**DOI:** 10.1186/s13073-023-01158-7

**Published:** 2023-02-14

**Authors:** Shumin Li, Bin Yan, Thomas K. T. Li, Jianliang Lu, Yifan Gu, Yueqiu Tan, Fei Gong, Tak-Wah Lam, Pingyuan Xie, Yuexuan Wang, Ge Lin, Ruibang Luo

**Affiliations:** 1 Department of Computer Science, The University of Hong Kong, Hong Kong, China; 2 Department of Obstetrics & Gynecology, Queen Mary Hospital, The University of Hong Kong, Hong Kong, China; 3 NHC Key Laboratory of Human Stem Cell and Reproductive Engineering, School of Basic Medical Science, Institute of Reproductive and Stem Cell Engineering, Central South University, Changsha, 410008 Hunan China; 4 Clinical Research Center for Reproduction and Genetics in Hunan Province, Reproductive and Genetic Hospital of CITIC-Xiangya, Changsha, 410013 Hunan China; 5 Hunan Normal University School of Medicine, Changsha, 410013 Hunan China; 6 National Engineering and Research Center of Human Stem Cell, Changsha, Hunan China; 7 College of Computer Science and Technology, Zhejiang University, Hangzhou, China

**Keywords:** Ultra-low-coverage whole genome sequencing, Imputation, Single-nucleotide polymorphisms, Genome-wide association study, Gestational age, Preterm birth

## Abstract

**Background:**

Very low-coverage (0.1 to 1×) whole genome sequencing (WGS) has become a promising and affordable approach to discover genomic variants of human populations for genome-wide association study (GWAS). To support genetic screening using preimplantation genetic testing (PGT) in a large population, the sequencing coverage goes below 0.1× to an ultra-low level. However, the feasibility and effectiveness of ultra-low-coverage WGS (ulcWGS) for GWAS remains undetermined.

**Methods:**

We built a pipeline to carry out analysis of ulcWGS data for GWAS. To examine its effectiveness, we benchmarked the accuracy of genotype imputation at the combination of different coverages below 0.1× and sample sizes from 2000 to 16,000, using 17,844 embryo PGT samples with approximately 0.04× average coverage and the standard Chinese sample HG005 with known genotypes. We then applied the imputed genotypes of 1744 transferred embryos who have gestational ages and complete follow-up records to GWAS.

**Results:**

The accuracy of genotype imputation under ultra-low coverage can be improved by increasing the sample size and applying a set of filters. From 1744 born embryos, we identified 11 genomic risk loci associated with gestational ages and 166 genes mapped to these loci according to positional, expression quantitative trait locus, and chromatin interaction strategies. Among these mapped genes, *CRHBP*, *ICAM1*, and *OXTR* were more frequently reported as preterm birth related. By joint analysis of gene expression data from previous studies, we constructed interrelationships of mainly *CRHBP*, *ICAM1*, *PLAGL1*, *DNMT1*, *CNTLN*, *DKK1*, and *EGR2* with preterm birth, infant disease, and breast cancer.

**Conclusions:**

This study not only demonstrates that ulcWGS could achieve relatively high accuracy of adequate genotype imputation and is capable of GWAS, but also provides insights into the associations between gestational age and genetic variations of the fetal embryos from Chinese population.

**Supplementary Information:**

The online version contains supplementary material available at 10.1186/s13073-023-01158-7.

## Background

Detection and characterization of genetic variants associated with traits and diseases are fundamental to the study of human genetics. Genome-wide association study (GWAS) is an approach widely used in genetic research that aims to decode the associations of specific genetic variations with particular diseases or traits in sample populations. In the past decade, GWAS has facilitated discovery of over one hundred thousand variants associated with complex traits in human [1]. Whole genome sequencing (WGS) has emerged as a dominant technology in GWAS because it enables one to generate a comprehensive view of the genomic variation landscape for not only a specific trait but also for common diseases. Thus, WGS-based approaches hold a significant advantage over genome-wide genotyping arrays or exome sequencing in the analysis of complete genetic variations. However, with a fixed budget, the high cost of sequencing many DNA samples is a limitation for GWAS [2–4]. Recently, to reduce the cost of sequencing, a number of low (0.5–1×) or extremely low-coverage (0.1–0.5×) WGS have been carried out as an alternative method of genotyping [2, 5, 6]. It is, however, unclear whether ultra-low-coverage WGS (ulcWGS) below 0.1× data can capture enriched genetic variations across the entire allele frequency (AF) spectrum. When considering a balance between number of samples sequenced and sequencing read coverage, effective genotype imputation could provide more authentic single-nucleotide variants (SNVs) that would be helpful for genetic research.

Genotype imputation can be used to infer missing genotypes and to increase the accuracy of detecting genetic variants, such as SNVs. In general, performance of genotype imputation is largely affected by sample size, sequencing coverage, analysis methods, and other parameters [7]. A main challenge to use very low-coverage WGS is how to achieve an adequately accurate imputation for downstream analyses. Previous attempts have shown the efficiency of low-coverage WGS, for example, a high r^2^ of imputation accuracy observed by using 10 low-coverage WGS (~0.5×) as compared to known genotypes [6]. Pasaniuc et al. reported that the GWAS signals obtained from using 909 whole-exome sequencing (~0.24×) are comparable to using genotyping array [2]. Gilly et al. found that more true association signals were identified by WGS (~1.0×) than the traditional array-based study [5]. Using ulcWGS (0.06×–0.1×) with 141,431 samples from a Chinese genomic study, the accuracy of imputed genotypes reached 0.71 [8]. Even though the distribution of genetic background from large number of samples is expected to compensate for the low sequencing coverages, it has never been determined how many samples are needed to achieve a relatively high accuracy. More importantly, lack of comparative data with coverages less than 0.05× results in the limited application of ulcWGS to GWAS.

Gestational age is an important complex trait associated with biological processes and human disease. Biologically, gestational duration plays a vital role in both mental and physical health of children at an age of 5 years old [9]. Gestational age shorter than 37 weeks is categorized as preterm birth (PTB). Previous studies found the contribution of both the maternal and fetal genomes to variation of gestational ages [10–12]. However, they focused on European and African samples by involving few samples from Chinese ancestries. Overall, biological mechanisms underlying variation of gestational durations remain unclear, primarily because insufficient maternal or fetal genotypes with widespread gestational ages have been collected [13]. Recently, preimplantation genetic testing (PGT) with trophectoderm biopsy for embryo aneuploidy screening has become a common practice in in vitro fertilization [14, 15], and poses as an expectant source of genotypes for GWAS. However, if the average sequencing coverage of PGT is even lower than the lowest levels that have been reported in GWAS so far, it is necessary to examine whether such PGT datasets are appropriately applied to GWAS.

In this study, we devised a pipeline for analyzing and applying the ulcWGS of 17,844 embryo samples for GWAS. Our result shows that a large sample size is effective to increase the accuracy of genotype imputation even at an ultra-low coverage. Furthermore, using the imputed genotypes of 1744 embryos that were successfully transferred and born with a widespread of gestational ages, we demonstrate the power of using ulcWGS in GWAS and provides insights into understanding genetic association of gestational age in embryos acquired from Chinese population. Refreshing the lowest coverage used in GWAS, our finding also provides a foundation for exploring the utilization of an even lower coverage for dissecting genotype-phenotype associations.

## Methods

### Samples and sequencing coverage

The whole PGT dataset of 17,844 embryos (1744 embryos in the dataset were transferred and given birth with complete clinical records) was from the Clinical Research Center for Reproduction and Genetics in Hunan Province, Reproductive and Genetic Hospital of China International Trust Investment Corporation - Xiangya. The samples used in this study were not used in any previous studies. The protocol of embryo culture and biopsy was published in a related study [16]. Three WGA kits were applied to the biopsied TE cells by following the manufacturer’s guides, including QIAGEN REPLI-g Mini Kit (called MDA), GenomePlex WGA4 Single Cell Whole Genome Amplification Kit (called dop-PCR), and Rubicon Genomics PicoPlex Single Cell Whole Genome Amplification Kit (called PicoPlex). A 1–2 μg of the WGA product was subjected to library construction and sequencing on the four platforms, including BGI-Seq 500, Illumina MiSeq, Ion Proton, Ion Torrent (Additional file 1: Table S2).

### Study design

**Fig. 1:**
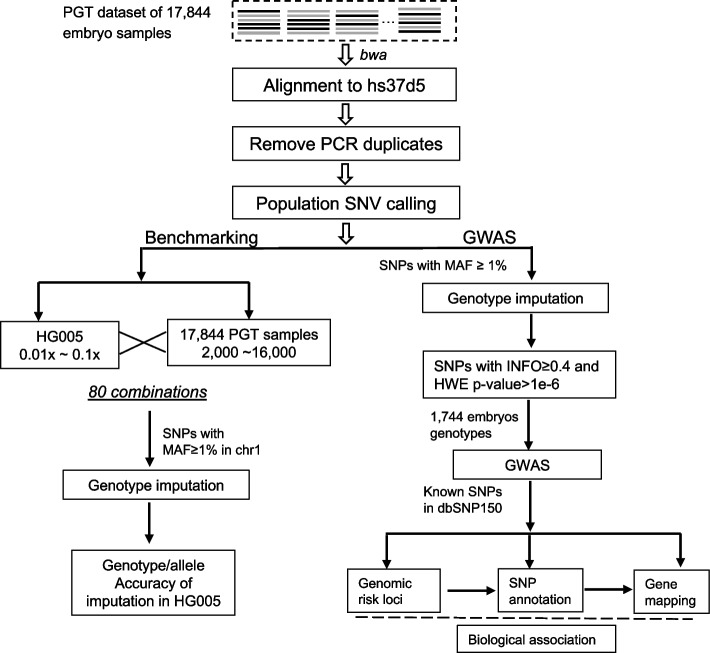
An overview of the analysis and benchmarking pipeline for ultra-low-coverage WGS

We developed a three-step pipeline to carry out genotype imputation using ulcWGS data and to perform GWAS (Fig. 1). Firstly, the raw reads of the 17,844 embryo samples were aligned to the hs37d5 reference genome. Sequencing coverage of these embryo samples displays a distribution with an average coverage 0.04× (Additional file 2: Fig. S1). To our best knowledge, it is below the coverage of any dataset used previously for genotype imputation. After removing potential PCR duplicates, the aligned reads were used to call population SNVs. Secondly, we conducted genotype imputation on each individual sample at the called population SNVs and assessed the genotyping accuracy based on the standard Chinese sample HG005 with known genotypes from GIAB (Genome in a Bottle, NIST) [17, 18]. Last, we applied the imputed genotypes from 1744 born embryos with complete follow-up records to GWAS and explored biological associations between the genetic variants detected in the born embryos and their gestational ages.

### Sequencing read processing and alignment

In the first step of Fig. 1, the raw reads of the PGT samples were delivered in two types, fastq or BAM. For BAM data, we used bedtools [19] to extract the raw reads into single-end fastq files. The raw reads of each sample were then aligned to the hs37d5 reference genome using BWA [20]. BWA-mem was applied to samples sequenced by the Ion Torrent with longer reads and “bwa aln” was used for the rest of the samples with shorter reads. Samtools rmdup [21] was used to remove the potential PCR duplicates.

### Population SNV calling

In the population SNV calling stage, we modified the method of Liu et al. [8]. The first stage is to use log-likelihood estimation for AF estimation, and the second stage is to use log-likelihood ratio test for determining allelic types. More details are described in Additional file 3: Supplementary Methods.

SNV calls of the raw population were filtered following the rules, (1) calls that overlapped with the 35-kmer problematic alignment regions in hs37d5 were removed [22, 23]; (2) calls that overlapped with regions with ENCODE mappability uniqueness score unequal to 1 were removed [24, 25] (tool “bigWigToBedGraph” used afterward to convert the bigwig into bed format) [26, 27].

### Genotype imputation

We used STITCH [28] version 1.5.7 for genotype imputation. The ancestral haplotypes number *k* were set as 20, the assumed number of generations *nGen* was set to 2000, and the reads were binned into windows with *gridWindowSize* 10000. The *diploid* mode in STITCH was used. Although using a reference panel is optional in STITCH, we used the IMPUTE2 1000 genome haplotypes phase 1 reference panel [29] as it improves the accuracy of imputation when the sample size is small. With sample size larger than 10,000, the improvement was not significant. We noted that the embryos used in the PGT were related family-based sibling samples according to the clinical protocol. Because our PGT data have ultra-low coverage (~0.04×), these related samples will monotonically increase the percentage of covered bases (Fig. S1d). Thus, the relatedness of the samples will have positive impact on the genotype imputation. All parameters were optimized by maximizing the *r*^2^ of the estimated AFs between imputation and population SNV calling in a randomly chosen 5 Mbp genomic region (chr3:180–185Mbp). When applying STITCH to the whole genome, we divided the genome into 5-Mbp windows with a 500-kbp overlap between two windows.

For benchmarking, seqtk was used to subsample the HG005 Illumina WGS raw reads to 0.01×–0.1× (Paired-end 250bp, 300-fold) [30]. We aligned the reads to hs37d5 by using the same pipeline as used in the embryo samples. Because a computer takes a few years to impute whole genome with tens of thousands of samples, we worked on only chromosome 1, the longest one in human. The genotype imputation was benchmarked according to 80 combinations of the 10-scale coverages of HG005 with 8 sizes of our samples from 2000 to 16,000, respectively.

All bi-allelic SNPs with MAF ≥ 0.01 found in chromosome 1 with the population SNV calling from the 17,844 samples are included for genotype imputation. We used the 167,814 SNPs both in our SNP callset and HG005 known genotypes for benchmarking. The imputed genotypes were compared to the truth released by GIAB for estimating the imputation accuracy. Then, genotype imputation was applied to all 17,844 embryo datasets at the 31,622,332 bi-allelic sites with MAF ≥ 0.01 found in population SNV calling. The entire imputation process spent 19 days and used 15 machines with 16 cores (two 8-core Intel Xeon Silver 4108 CPU). Two filters “INFO score ≥ 0.4 and HWE *p-*value >1e−6” were applied to select the imputed genotypes.

### Genome-wide association study (GWAS)

To conduct GWAS, we used score statistics [31] that is implemented in ANGSD [32]. Variants satisfying four conditions were selected as inputs, including (1) known in dbSNP150, (2) MAF ≥ 0.01, (3) INFO score ≥ 0.4, and (4) HWE *p*-value > 1e−6. To remove biases, we specified 16 covariates for ANGSD, 8 most significant principal components calculated from the inputs of PCA (Additional file 3: Supplementary Methods), and 8 clinical records including maternal age, maternal BMI, fetal sex, either parent with single-gene disease, either parent with chromosome abnormality, multiple pregnancy, preeclampsia, and gestational diabetes of mellitus. Except the default parameters, we set *minHigh* to 15 (requiring at least 15 high credible genotypes from the input) instead of 10 to achieve better accuracy with a large sample size. ANGSD did not create an output beta-coefficient, so we followed the ideas of Skotte et al. [31] by incorporating the genotype probabilities and all 16 covariates into a linear regression model, with the gestational age as a response variable. The effect size was calculated by the coefficient of genotypes.

### Independent SNPs and genomic risk loci

The significant SNPs from our GWAS were mapped to genomic risk loci using FUMA pipeline [33] and the LD information of 1000G EAS variants [34]. We first defined “independent significant SNP,” a SNP that meets genome-wide significance level (*p*-value ≤ 4.515e−8) and is independent of other significant SNPs (with LD *r*^2^ < 0.6). FUMA also generated a set of lead SNPs with low LD and with other (*r*^2^ < 0.1) from the independent significant SNPs. The genomic risk loci were identified by starting from these lead SNPs and through iteratively merging related genomic regions to them according to FUMA’s rules.

Also, the FUMA pipeline sorted out a set of candidate SNPs from our inputs that meets one of two conditions, (1) the independent significant SNPs and (2) SNPs that are linked to the independent significant SNPs (with LD *r*^2^ ≥ 0.6). For condition 2, the SNPs can be from our imputed genotypes if *p*-value is below 0.05 or from the reference panel of 1000G EAS. ANNOVAR was used to annotate the candidate SNPs [35].

### Functional annotation of the mapped genes

DAVID online tool [36] was used to analyze the enrichment of Gene Ontology (GO) biological processes and KEGG pathways for the coding genes mapped to the risk loci.

### Gene mapping

We used three gene-mapping strategies provided by FUMA [33], including positional, expression quantitative trait locus (eQTL) and chromatin interaction. For positional mapping, ANNOVAR annotations were used. The candidate SNPs were mapped to the nearest genes within a maximum 10-kbp distance. For eQTL mapping, expression data of all tissue types in GTEv6, GTEv7, and GTEv8 [37] were used. We required false discovery rate (FDR) < 0.05 and *p*-value <0.001 for a valid eQTL mapping. All chromatin interaction data in FUMA were used [38–41]. The promoter was set to upstream 2000 bp to downstream 500 bp of transcriptional starting sites. We required FDR < 1e−6 for a valid chromatin interaction mapping.

### Analysis of genome-wide mRNA expression data

We first extracted the genome-wide microarray and RNA-seq data of human mRNA expression from GEO database [42]. The mRNA data includes maternal PTB, infant PTB, infant disease, and breast cancer (Additional file 1: Table S8). Based on the normalized expression data provided by the database, we analyzed differentially expressed genes (DEGs) between different conditions, including (1) PTB vs. normal term, (2) BPD or sepsis vs. infant without BPD or sepsis, and (3) breast cancer vs. control samples. For microarray platform-based data, we used the *limma* package in R programming language and conducted empirical Bayes moderated *t*-test [43]. DEGs were detected with a fold change above 1.5 and *p*-value below 0.05. For RNA-seq data with raw counts, we utilized the edgeR method to identify DEGs [44]. The DEGs are listed in Additional file 1: Table S9.

## Results

### Benchmarking genotype imputation using the ultra-low-coverage sequencing data of 17,844 embryos and HG005

**Fig. 2:**
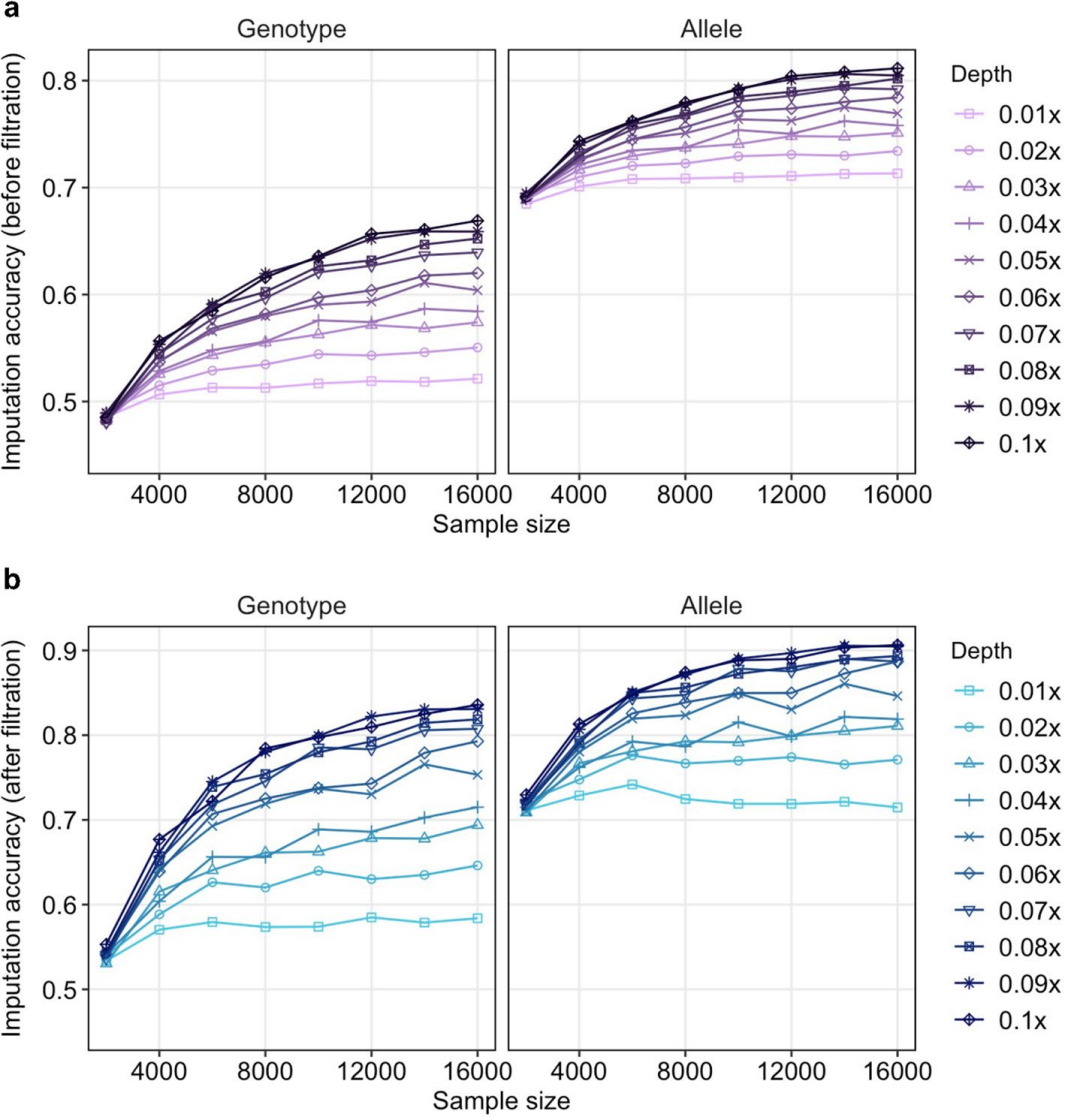
Imputation accuracy at different coverages and sample sizes. The accuracies of imputed genotype or allele were obtained by comparing with the known genotypes in HG005 (**a**). After using filter “INFO score ≥ 0.4 and HWE *p*-value > 1e−6”, the accuracies of imputed genotype or allele (**b**)

We estimated the accuracy of the imputed genotypes from the SNPs of chromosome 1 both called in our 17,844 samples and the known genotypes in HG005. The genotype imputation was benchmarked according to 80 combinations of the 10-scale coverages of HG005 with 8 sizes of our samples. A monotonic increase in accuracy with sequencing coverage was observed (Fig. 2a), consistent with previous studies [2, 28]. The accuracy for sample size of 2000 stayed at around 0.48 under all sequencing coverages. But for a larger sample size of 16,000, its accuracy increased from 0.48 at 0.01× to 0.66 at 0.1×. This result suggests that at ultra-low coverages, increase in sample size could obtain higher accuracy (Additional file 1: Table S1). In general, a lower coverage with a larger sample size results in better performance than a higher coverage with a smaller sample size. For example, the genotype accuracy at 0.05× with 14,000 samples versus 0.1× with 4000 embryos was 0.61 versus 0.55. A larger sample number is therefore more efficient in optimizing genotype imputation than increasing sequence coverage. It is also noticed that at the two lowest coverages in our experiments, the contribution of increasing sample size was not significant and the accuracy plateaued at 0.52 (0.01×) and 0.55 (0.02×). Using the same datasets, we evaluated allele accuracy that relaxed zygosity correctness from genotype accuracy. The corresponding accuracies were much better (increased to 0.7 and higher) while maintaining the same trend with increasing sample size and coverage (Fig. 2b). Therefore, when the genotypes are incorrectly imputed for some SNPs, the non-reference allele could be correctly detected.

**Fig. 3:**
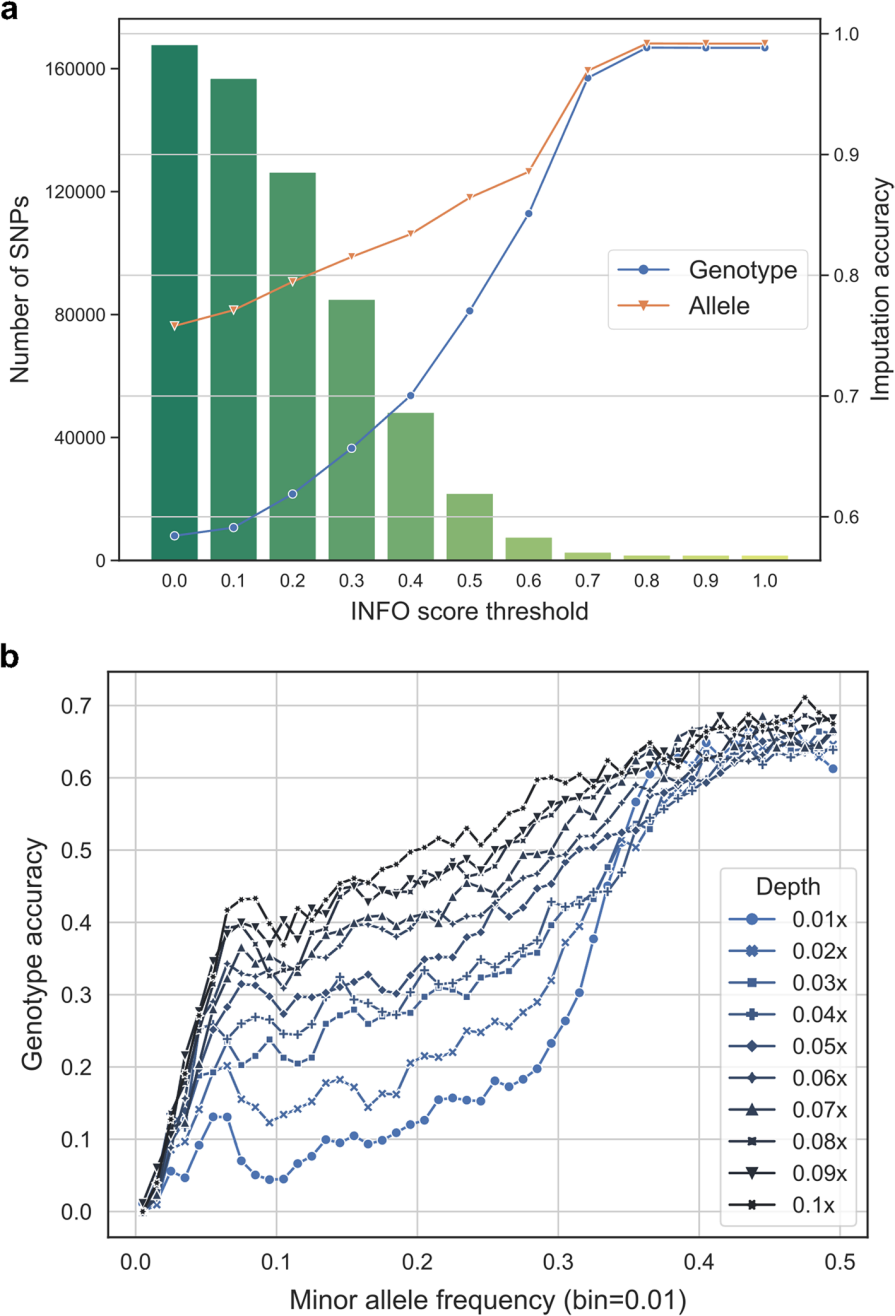
Performance of different imputation result filters. The accuracies of our samples were calculated against the known genotypes in HG005. **a** Effect of INFO score filtering cutoffs on genotype and allele accuracies. The imputation was conducted by using 0.04× sequencing coverage of HG005 and with 16,000 embryo samples. **b** Effect of MAF bins on genotype accuracy at multiple sequencing coverages. The imputation was conducted through different sequencing coverages of HG005 with 16,000 embryo samples

We next examined several important quality metrics that are widely used to filter falsely imputed genotypes. The INFO score of IMPUTE2 style [45] denotes the certainty of an imputed genotype and has been accepted as a quality metric of imputation. For a combination of 0.04× coverage with 16,000 samples, we benchmarked ten different INFO score thresholds from 0.1 to 1.0 and detected corresponding SNPs. As increase in the INFO scores, we observed a consistent increase in genotype accuracy from ~0.60 to 0.99, but a rapid decrease in number of SNPs that meet these thresholds (Fig. 3a). Thus, INFO score could act as an effective metric to evaluate the accuracies at ultra-low coverage, but its thresholds should be meticulously chosen in order to retain sufficient SNPs. Effect of MAF scores on genotype accuracy of HG005 was then tested as a potential metric. We divided the genotyping results at different sequencing coverages (sample size fixed to 16,000) into bins of MAF ranges (0.01 MAF a bin) and calculated the genotype accuracy each bin. The genotype accuracy increased rapidly from MAF 0 to 0.05 and reached a turning point at 0.05. After this point, the accuracy became slow increasing (Fig. 3b). However, even for the most common SNPs (MAF 0.4~0.5), the accuracy was converged at ~70%. The accuracy of the two lowest coverages 0.01× and 0.02× fluctuated especially at low MAF cutoffs. Because such fluctuation was not observed during INFO testing, MAF might not be a reliable metric to change genotype accuracy at ultra-low coverage. In subsequent analyses, we followed the common practice to use SNPs with MAF ≥ 0.01 for GWAS. Finally, we combined HWE *p*-values with INFO scores as a filter but without losing too many SNPs. HWE *p*-values could evaluate the probability of the imputed genotype at a certain SNP that is significantly different from the expectations by HWE. We summarized the genotype accuracy of different combinations of INFO scores and HWE *p*-value cutoffs in Table 1. When INFO scores were set above 0.4, the accuracies of genotype and allele were 70.0% and 83.4%, respectively, with 48,176 SNPs left. The “INFO score ≥ 0.4 and HWE *p*-value >1e−6” resulted in an increased accuracy 71.5%, with 28,773 SNPs left. Thus, our GWAS utilized this setting, “INFO score ≥ 0.4 and HWE *p*-value >1e−6” as filtering criteria.

**Table 1:** Genotype imputation performance with different filtering criteria

Filtering criteria	SNP number	Genotype accuracy	Allele accuracy
None	167,814	0.584	0.758
Known SNP in 1000G reference panel	141,161	0.578	0.751
INFO score ≥ 0.1	156,835	0.591	0.771
INFO score ≥ 0.2	126,331	0.618	0.794
INFO score ≥ 0.3	84,932	0.656	0.815
INFO score ≥ 0.4	48,176	0.7	0.834
MAF ≥ 0.05	162,788	0.597	0.777
HWE *p*-value > 1e−9	84,237	0.595	0.695
HWE *p*-value > 1e−6	77,419	0.596	0.692
HWE *p*-value > 1e−3	65,561	0.599	0.687
HWE *p*-value > 1e−6 and INFO score ≥ 0.1	68,505	0.610	0.713
HWE *p*-value > 1e−6 and INFO score ≥ 0.2	55,972	0.646	0.751
HWE *p*-value > 1e−6 and INFO score ≥ 0.3	42,202	0.681	0.786
HWE *p*-value > 1e−6 and INFO score ≥ 0.4	28,773	0.715	0.818

Totally 16,000 samples with an average of 0.04× sequencing coverage were used for imputation

To summarize our benchmarking results for future study on ulcWGS, we built a regression model (Eq. 1) to calculate the expected genotype accuracy using sequencing coverage and sample size as inputs.

1 $$acc=2.227\times c+8.937{e}^{-6}\times s+0.494\ \left(c\ge 0.01,s\ge 4000\right)$$

where *acc* is the expected genotype accuracy, *c* denotes the sequencing coverage, and *s* denotes the sample size. The model has a *r*^2^ of 0.874 (Additional file 2: Fig. S2).

### GWAS of gestational ages using 1744 born embryos

With the solid foundation laid out in the previous section, we have obtained sufficient good-quality SNPs for GWAS. Among the 17,844 sequenced embryos, 1744 were transferred and gave birth to a baby. The gestational age of all 1744 born embryos are well documented and thus were chosen for biological associated study. We revised the population SNV calling method used in Liu et al. [8]. A total of 151,793,444 SNVs were detected and of 141,718,305 are bi-allelic. The MAF spectrum bi-allelic novel and known variants in dbSNPv150 [46], 1000G [34], and gnomAD [47] is shown in Additional file 2: Fig. S3a. The transitions/transversions ratios for “all bi-allelic SNVs” and “bi-allelic SNVs known in dbSNPv150” were 3.05 and 3.58, respectively. The Pearson correlation coefficient of the non-reference AFs between the 301 Chinese samples in 1,000G (so-called 1000G CHN [34]) and the corresponding SNVs obtained in our dataset was 0.986 (Additional file 2: Fig. S3b). This result supports a strong correlation between the two datasets, and high confidence of the known variants used in our analysis. We also performed genotype imputation at bi-allelic population SNPs with MAF ≥ 0.01. The Pearson correlation coefficient was 0.985, showing a high consistency between the estimated AFs in population SNV calling and in imputation.

Three different whole genome amplification (WGA) methods and four different sequencing platforms were used in the PGT dataset (Additional file 1: Table S2). It is not uncommon that large number of samples may use multiple sequencing platforms and WGA. Removing these unrelated covariates from GWAS as much as possible is essential especially when the sequencing coverage is ultra-low. Such covariates should be detected and disregarded in GWAS. We applied principal component analysis (PCA) to the imputed genotypes of SNPs with MAF ≥ 0.05 among all 17,844 embryo samples (Additional file 3: Supplementary Methods, Additional file 2: Fig. S4a). The first and second principal components distinguish the differences of sequencing platforms (Additional file 2: Fig. S4b) and of WGA methods (Additional file 2: Fig. S4c). Therefore, we used the top eight principal components and eight other clinical records as covariates in GWAS. PCA was also applied to the GWAS samples and the top principal components were included in the subsequent analyses as covariates for removing the biases.

We used the state-of-the-art one-stage GWAS strategy [48] to analyze the 1,107,198 imputed SNPs in the 1744 transferred and born embryo samples with complete follow-up records. The distribution of gestational ages shown in Additional file 2: Fig. S5 include 162 preterm deliveries (gestational age < 37 weeks), 42 early preterm deliveries (gestational age <34 weeks), and 8 very early preterm deliveries (gestational age <28 weeks). The gestational ages were standardized by *z*-score and incorporated as a quantitative trait.

**Fig. 4:**
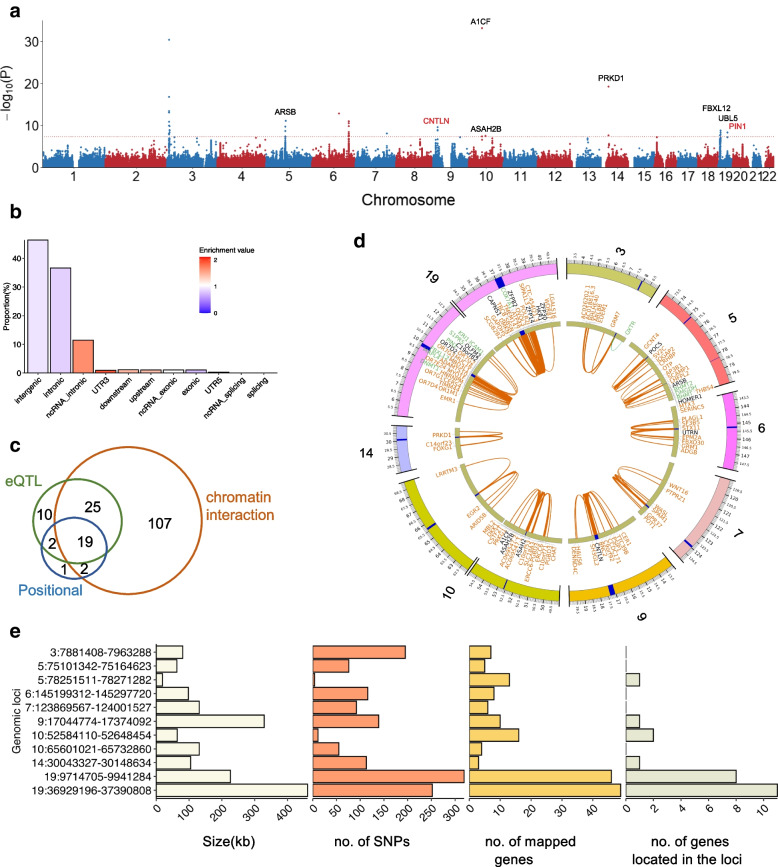
SNP-based genome-wide association on gestational age. **a** Manhattan plot of the SNPs in GWAS. The red dash line represents the genome-wide significance level 4.515e−8. The SNP “rs946934582” with *p*-value of 2.764e−144 is beyond the scale, thus hereby listed alone. The genes shown are linking with the candidate SNPs and position of the corresponding genomic risk loci. **b** Functional annotation and enrichment test result of the candidate SNPs in FUMA. **c** A Venn diagram of the 166 genes that could be mapped to the 11 genomic risk loci by positional, eQTL and chromatin interaction strategies. **d** A Circos plot of the chromatin interactions and eQTL mapping in the 11 genomic risk loci from eight chromosomes. The outer ring is chromosomes, the regions in blue denote genomic risk loci. The middle ring represents the mapped genes. The color of the gene symbols shows how they were mapped, eQTL in green, chromatin interaction in orange, and both eQTL and chromatin interaction in black. The inner ring shows the linking edges, eQTL in green, and chromatin interaction in orange. **e** A summary of the 11 genomic risk loci

A total 1,107, 198 SNPs with imputed genotypes were selected for GWAS that are in accord with the following: (1) MAF ≥ 0.01, (2) known in dbSNPv150, and (3) passed the filter “INFO score ≥ 0.4 and HWE *p*-value >1e−6”. The Q-Q plot shows a large deviation of the observed *p*-values from the null hypothesis (Additional file 2: Fig. S6). The linkage disequilibrium score regression (LDSC) software package [49] with 1000G EAS reference was used to estimate *λ*_*GC*_ = 0.992, mean *χ*^2^ = 1.012. The LD score regression intercept was 0.952, standard error = 0.021, indicating that the population stratification and other factors were well-controlled. We identified 40 significant SNPs satisfying Bonferroni-corrected significant levels of 4.515e−8. The Manhattan plot shows the distribution of the detected SNPs cross all chromosomes (Fig. 4a, Additional file 1: Table S3).

We used FUMA [33] pipeline for GWAS downstream analysis. First, FUMA generated a set of candidate SNPs and the 11 independent SNPs (Additional file 1: Table S4). By annotation, most of the SNPs are located and enriched in intergenic and intronic regions (Fig. 4b), which is similar to the previous study [50]. Specifically, there are 11 SNPs located in exons (0.8% of total), and 4 of them are nonsynonymous SNPs (Additional file 1: Table S5). Additionally, we observed the distribution of regulatory elements and chromatin states with the candidate SNPs (Additional file 2: Fig. S7). According to RegulomeDB scores assigned to each candidate SNP, 1.09% SNPs were classified as likely to affect regulator binding (score 2a and 2b) and 0.21% as likely to affect regulator binding and linked to expression of a gene target (score 1d and 1f). These proportions of SNPs hold a relatively high likelihood to affect the regulatory elements along noncoding regions. Second, we identified 11 leading SNPs from their corresponding genomic risk loci by FUMA. Figure 4e shows the DNA length, number of SNPs, and mapped genes of these risk loci. The zoom in locus plot of the 11 risk loci are shown in Additional file 2: Fig. S8. Four risk loci were reported to be associated with refractive astigmatism, adolescent idiopathic scoliosis, glomerular filtration rate, among others, indicating a possible connection of these diseases or traits with PTB (Additional file 1: Table S6).

By integrating strategies positional, eQTL, and chromatin interaction mappings, we identified a total of 166 genes mapped to the 11 risk loci, including 48 and 19 genes from two and three strategies, respectively (Fig. 4c, Additional file 1: Table S7). There were 24 genes detected within or less than 10 kbp from the candidate SNPs and 7 of them are shown in Fig. 4a based on the location of the genomic risk loci. Importantly, *CNTLN* was reported as a PTB-related gene [51], and *PIN1* involves inhibition of breast cancer [52]. A Circos plot shows the graphic distribution of the mapped genes via eQTL and chromatin interaction, and their links with the genomic risk loci (Fig. 4d). The breakdown of each chromosome is shown in Additional file 2: Fig. S9. Even though not within any risk loci, *CRHBP* was linked through chromatin interaction mapping to two loci, chr5:75101342-75164623 and chr5:78251511-78271282. Enrichment analysis of DEGs in 30 tissue types in GTEx v8 [53] exhibits significant overexpression of the mapped genes in both ovary and uterus (Additional file 2: Fig. S10a). The gene set enrichment analysis also indicates their association with the immune system, breast cancer, and transcriptional regulation (Additional file 2: Fig. S10b).

### Association of the 166 mapped genes from GWAS with preterm birth, infant disease, and breast cancer

**Fig. 5:**
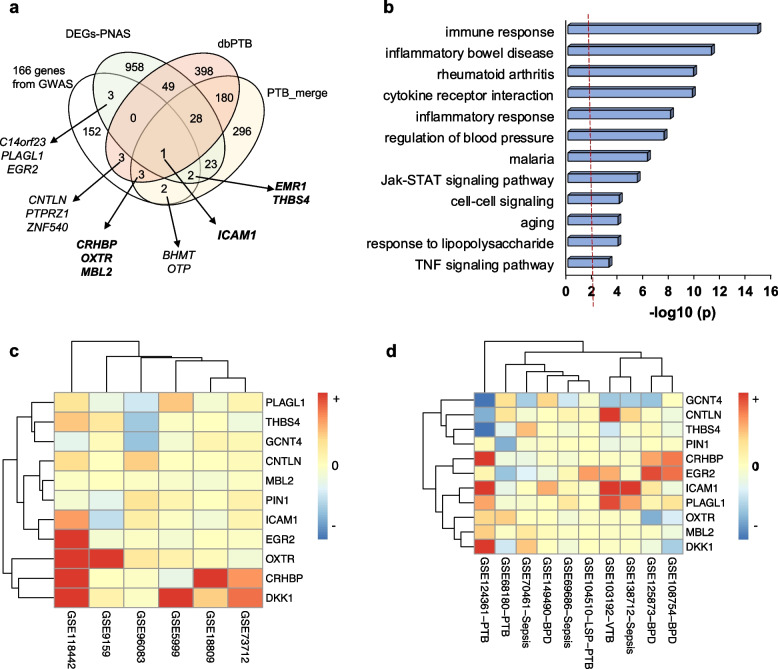
Comparison of the 166 genes mapped to 11 genomic risk loci with PTB and infant disease. **a** The 166 mapped genes were compared with 3 sets of reported PTB genes including dbPTB from Sheikh et al. [57] and Uzun et al. [58], PTB-merged from 5 resources [10], and PNAS-identified DEGs in 2019 [10] (see Additional file 1: Table S7). **b** A bar graph showing significantly enriched GO biological processes and KEGG pathways based on the 50 PTB marker genes (see Additional file 1: Table S7). **c,d** Heatmaps showing expression profiling and clusters of the PTB genes predicted from GWAS under maternal PTB (**c**) and PTB infant with BPD and sepsis (**d**). The gene expression data was extracted from the listed GSE accession numbers of NCBI/GEO

GWAS of gestational age related PTB has been implicated in biological functions that include immune response, inflammatory response, and coagulation factors [11, 54–56]. We compared our 166 mapped genes with reported PTB markers by collection of 8 published resources, here classed to 3 PTB sets, including dbPTB from Sheikh et al. [57] and Uzun et al. [58], PTB-merged from 5 data resources [10], and PNAS-identified DEGs of PTB in 2019 [10]. We found that *CRHBP*, *EMR1*, *ICAM1*, *MBL2*, *OXTR*, and *THBS4* have been reported in at least 2 PTB sets. Specifically, *ICAM1* was present in all 3 sets and 6 data resources, and both *CRHBP* and *OXTR* in 5 resources (Additional file 1: Table S7). In addition, there were 8 genes overlapped with 1 PTB set (Fig. 5a). This result pinpoints a relationship of the detected risk loci with PTB, and possible roles of the overlapped genes in PTB. There are totally 1930 genes reported as PTB-related; however, only 50 were frequently recognized by at least 5 data resources, hereafter referred to as PTB marker genes (Additional file 1: Table S7). The 50-PTB marker set was significantly enriched with inflammatory and immune response-related processes or pathways (Fig. 5b). Similarly, those PTB genes that overlapped with 3 or 4 resources mainly participate in the same biological functions. The PTB-related genes listed at Fig. 5a involve immune response (*EMR1*, *ICAM1*, *PTPRZ1*, and *MBL2*), inflammatory response (*CRHBP*, *ICAM1*, *PTPRZ1*, and *MBL2*), coagulation (*MBL2*), apoptosis (*PLAGL1*), and cell adhesion (*ICAM1* and *THBS4*), emphasizing their associations with PTB.

To determine the relationship between the mapped genes and PTB, we first analyzed the DEGs between maternal PTB and normal term birth based on six published datasets of genome-wide gene expression (Additional file 1: Tables S8 and S9). Significant overexpression of *CRHBP* and *OXTR* were identified in two datasets, while overexpressed (*ICAM1*, *EGR2*, and *PLAGL1*) and underexpressed (*THBS4*) genes were present in one dataset (Table 2). Another *DKK1* expressed higher levels above 2.0-fold in two datasets, suggesting its importance in PTB. Then, we analyzed four infant PTB datasets of gene expression and found differentially increased expression of *ICAM1*, *CRHBP*, *DKK1*, *EGR2*, and *PLAGL1*, consistent with maternal PTB. In contrast, significantly underexpression of *THBS4*, *PIN1*, and *GCNT4* was commonly detected by maternal and infant subgroups (Table 2). It is also noticed that *DNMT1* shows increased expression in both fetal and maternal groups (Table 2). DNA methylation was suggested to involve generation of early PTB [10]; however, the role of *DNMT1* as a DNA methyltransferase in PTB has not been determined yet. This analysis provides evidence that these mapped DEGs above including *DNMT1* are associated with both maternal and infant PTB. Relatively, *OXTR* seems to be related to only maternal factor. A heatmap of mRNA expression shows clustering of 6 expression profile cross PTB and normal term conditions (Fig. 5c). Two clusters display distinct expression patterns of these PTB genes.

**Table 2:** Main mapped genes differentially expressed in PTB, infant disease, and breast cancer

Genes mapped to risk loci	Overlapped no. with the reported PTB datasets	No. of DEGs detected in gene expression data						
		Maternal PTB	Infant PTB	Infant BPD	Infant sepsis	Breast cancer ER, PR	Breast cancer TNBC	Breast cancer HER2+
*ICAM1*	6	1(up)	2(up)	1(up)	1(up)	1(up),2(down)	2(up)	1(up)
*CRHBP*	5	2(up)	1(up)	1(up)				
*OXTR*	5	2(up)				1(up)	1(down)	
*THBS4*	3	1(down)	1(down)		1(up)	1(up)	2(down)	1(up),1(down)
*EGR2*	2	1(up)	1(up)	1(up)		1(up)	2(down)	2(down)
*CNTLN*	1	1(up)	1(up),1(down)		1(up)	1(down)		1(down)
*MBL2*	4		1(up)					
*EMR1*	2			1(up)				
*PLAGL1*	1	1(up)	2(up)		1(up)	2(down)	2(down)	2(down)
*DKK1*		2(up)	1(up)	1(down)		2(down)	3(up),1(down)	1(up),1(down)
*GCNT4*		1(down)	2(down)	1(down)	2(down)	1(up),2(down)	1(down)	1(down)
*DNMT1*		1(up)	1(up)				1(up)	1(up)
*PIN1*		1(down)	1(down)					

PTB-related genes identified in our GWAS were compared with DEGs identified in published gene expression datasets (Additional file 1: Table S9). “up” and “down” indicate differentially overexpressed and underexpressed gene, respectively

It is well known that bronchopulmonary dysplasia (BPD) is the most common respiratory disorder among children born preterm [59, 60]. The pathogenesis of BPD involves multiple prenatal and postnatal mechanisms affecting the development of immature lung. Also, neonatal sepsis is associated with severe morbidity and mortality during the neonatal stage. The incidence of late-onset sepsis increases with increase in survival rate of preterm and low weight babies [61]. Thus, we examined the possible relationship of the 166 genes with infant BPD and sepsis by analyzing gene expression data derived from samples of preterm infants (Additional file 1: Table S8). We first identified differentially increased expression of *CRHBP*, *ICAM1*, and *EGR2* under PTB of maternal and infant and BPD conditions, but differentially decreased expression of *DKK1* (Table 2). Under sepsis condition, differentially overexpressed *CNTLN*, *ICAM1*, and *PLAGL1* in PTB were consistently observed. By contrast, *GCNT4* expression was always significantly decreased under maternal and infant subpopulations. The gene clustering shows diversity of gene expression across different samples with infant PTB; however, these upregulated genes were grouped together (Fig. 5d), supporting the idea that BPD or sepsis induces the change in these PTB genes.

Breast cancer is one of the most frequently diagnosed malignancies observed during pregnancy. It often presents characteristics of high malignancy and is hormone receptor negative like Estrogen receptor (ER)−, HER2+, or triple-negative breast cancer (TNBC). We collected gene expression data mainly presenting three subtypes of breast cancer, TNBC, HER2+, and ER or progesterone receptor (PR) (Additional file 1: Table S8). By analysis of DEGs, we detected the expression of PTB-related genes *DKK1*, *ICAM1*, *DKK1*, *EGR2*, *PLAGL1*, *GCNT4*, and *THBS4* in all three subtypes. Increased *ICAM1* and decreased *PLAGL1* were consistently identified in these datasets (Table 2). In fact, *ICAM1* has been reported as TNBC markers [62] and acts as prognostic molecule of breast cancer [63]. Both *ICAM1* and *DKK1* could increase expression in TNBC cells [64]. However, other PTB-related genes do not display similar changes in expression under the cancer subtypes. For example, *OXTR* was detected by only one dataset of ER+/−, while *CRHBP*, *EMR1*, *PIN1*, and *MBL2* were not found among any of the subtypes. Conversely, underexpression of *PLAGL1* and *GCNT4* were found in all three types of breast cancer. In addition, overexpression of *DNMT1* was observed in TNBC and HER2+ subtypes that validates its oncogenic roles in breast cancer and drug target of TNBC [65, 66].

**Fig. 6:**
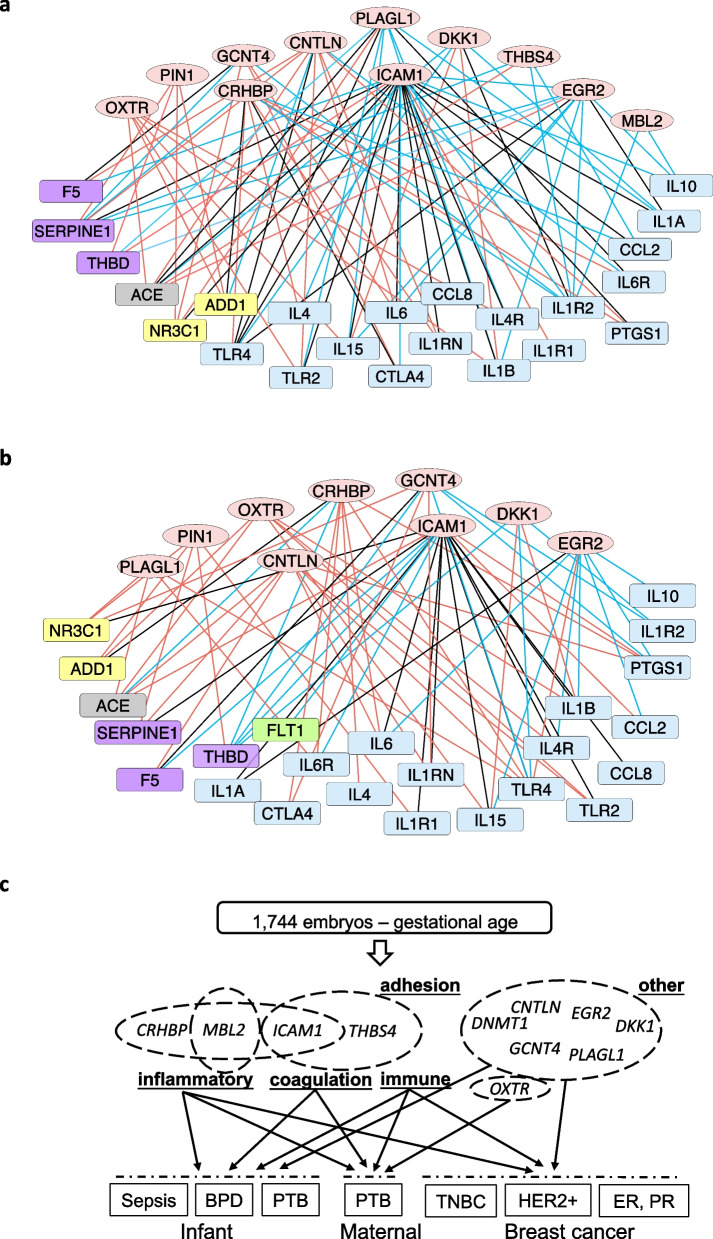
Associated analysis of the PTB-related genes in maternal and infant subtypes. **a** A gene co-expression network merging maternal and infant PTB subtypes. **b** A gene co-expression network merging maternal PTB and BPD of preterm infant. In **a** and **b**, oval nodes represent PTB-related genes predicted from GWAS using the 1744 born embryo samples, rectangle nodes refer to the 50 top PTB markers summarized from previously studies (Additional file 1: Table S7), involving immune or inflammation (blue), apoptosis (yellow), angiogenesis (green), coagulation (purple), and other biological processes (gray). Co-expressive edges (the Pearson correlation *p*-value < 0.01) linking nodes represent maternal (red), infant (blue), and both maternal and infant (black). **c** A graphic summary to illustrate gestational age’s association with PTB, infant disease, and breast cancer

To identify further interactions between the selected PTB genes from Table 2 and the top 50 PTB markers, we calculated Pearson correlation coefficient by comparing their gene expression of maternal and infant PTB, BPD, and sepsis subsets, respectively. We built the corresponding co-expression networks of the PTB genes (Additional file 2: Fig. S11). Clearly, the co-expressed genes involve immune and inflammatory responses, coagulation, and apoptosis and angiogenesis, among others. We then constructed the networks cross different subpopulations. As shown in Fig. 6a, the PTB genes could involve both maternal and infant PTB processes, especially *ICAM1*, *PLAGL1*, *EGR2*, and *CRHBP* that link to TLR4, a known preterm marker associated with immune and inflammatory processes [67]. Similarly, co-expression interactions of these genes with the top PTB markers were observed under maternal PTB and infant BPD (Fig. 6b). Both *OXTR* and *PIN1* only display gene correlations under maternal PTB, whereas *MBL2* only correlates under infant PTB. This analysis indicates that the predicted PTB-related genes including *ICAM1*, *CRHBP*, *PLAGL1*, *EGR2*, *CNTLN*, and *DKK1* play an important role in preforming biological activities associated with PTB, infant disease, and possibly breast cancer, due to the gestational age-induced.

## Discussion

Low- or very low-coverage sequencing data have been increasingly used in discovery of genetic variation and GWAS. However, if the coverage is further decreased to below 0.1×, how many samples are needed in imputation for obtaining a satisfying genotype accuracy and can ultra-low-coverage samples be properly applied to GWAS? To address this challenge, we used a PGT dataset of 17,844 embryo samples with an average of 0.04× coverage and achieved satisfying genotype imputation performance comparable. We first demonstrate that increasing in number of ulcWGS samples is more efficient than changing sequencing coverages and indicates the ability of such ultra-low-coverage PGT samples to obtain adequate accuracy of genotypes. Furthermore, we found that INFO score and HWE *p*-value are effective to act as filters to improve the accuracy at ultra-low coverages while meanwhile keeping enough SNPs for downstream analyses. In addition, it is noted that the biases induced by different sequencing platforms and WGA kits exist in ulcWGS data. To eliminate these biases, we selected the top PCs as their representations which were substantially incorporated as covariates in GWAS. Our results show the potential for this approach in processing similar effects derived from other PGT data. To our best knowledge, the samples used in this study hold the lowest average WGS coverage for GWAS so far, and our study provides a framework for guiding other researchers who work on ulcWGS data.

Gestational age is a multi-factor phenotype involved in maternal and fetal biological activities [68]. To investigate effects of the fetal genome on gestational age, we used the imputed genotypes of 1744 born embryo samples, a cohort of samples who successfully gave birth after in vitro fertilization. From the 166 genes mapped to the 11 genomic risk loci, we identified a set of the PTB-related genes that were previously reported [12, 68]. *CRHBP*, *ICAM1*, and *OXTR* are primary representative genes showing evidence of genetic association with gestational age among our samples. *CRHBP* is an important gene in maternal and fetal gestation that could regulate the pregnancy length by increasing/decreasing the concentration of CRH [69, 70]. *ICAM1* involves disease induced PTB during pregnancy [71–73]. Oxytocin signaling is mediated by oxytocin receptor (*OXTR*), which is related to gestational age [74]. We validated these predicted PTB genes by analysis of DEGs from maternal and infant PTB subpopulations.

PTB is neonatal birth occurring before 37 weeks of gestation age and is a leading cause of infant morbidity and mortality. Understanding of genetic and molecular mechanisms of PTB and its association with gestation duration is currently insufficient. Recently, Zhang et al. reported replicable loci in six genes (*EBF1*, *EEFSEC*, *AGTR2*, *WNT4*, *ADCY5*, and *RAP2C*) associated with gestational duration [12]. This study identified 3 genes (*EBF1*, *EEFSEC*, and *AGTR2*) strongly associated with PTB in a European ancestry cohort of 43,568 women. However, we did not detect such 6 genes from the reported PTB markers and only identified them as DEGs from few published datasets of PTB. This could be due to the heterogeneous sources of samples used in different PTB studies or be explained in part by the genetic complexity of incomplete gestation-induced PTB complications. Here, we collected almost 2000 PTB-related genes from previous PTB studies. The differences in study design, source and subtype of samples, and statistical methods would be important factors that could account for the diversity of PTB variants and genes among the various studies. Considering the possible association of PTB with other traits, we compared differential gene programs on multiple phenotypes between mother and infant reported in previous studies, and demonstrated a high risk of *CRHBP*, *ICAM1*, *DNMT1*, *CNTLN*, *PLAGL1*, *DKK1*, and *EGR2* with PTB among our sample cohorts of Chinese ancestry. To support this finding, we reconstructed co-expressive networks linking the PTB-related genes in GWAS with the reported PTB markers. Indeed, the correlated PTB genes are mainly involved in immune and inflammation-related processes and signaling pathways, as well as coagulation factors. Thus, these findings have biological implications for dissecting genetic associations of gestational factors with disease or traits in different human ancestries.

Pregnancy is possibly associated with an increased risk of developing breast cancer [75]. Although several reports indirectly described relationship between breast cancer and PTB [76], evidence is still lacking. To determine whether a correlation exists between PTB women and breast cancer pathogenesis, we compared differential expression of the PTB-related genes in PTB samples and three subtypes of hormone receptor-related breast cancer samples. Although several DEGs were found in both PTB and cancer subtypes, their expression patterns are not consistent. It perhaps is because of different subtype of samples derived from heterogeneous populations, as well as different phenotypes involved in the analyses. Nevertheless, our data provides additional evidence that PTB might be related with breast cancer hormone-related subtypes. A graphic overview of our results is summarized in Fig. 6c. We proposed interplays of gestational age with PTB, fetal disease, and breast cancer. The representative PTB genes *CRHBP*, *ICAM1*, *THBS4*, *DNMT1*, *CNTLN*, *PLAGL1*, *DKK1*, and *EGR2* are likely associated with these phenotypes by targeting immune and inflammatory response, coagulation, and cell adhesion.

GWAS proved useful in past decades in identifying phenotype-genotype associations, but the findings were not causal factors [77, 78]. Our findings provide insight into understanding the genetic basis of gestational age. The reported risk factors are not supported for clinical decision-making before more validated supporting data are provided. There are three possible reasons. First, gestational age is a complex trait involving genetic and environmental factors. Even if some non-genetic factors are included, additional variables are related to PTB, such as nutrition, physical activity, and psychological factors [79]. Applying the results to the risk profiling could lead to inaccurate predictions because of potential ignorance of gene-environment interactions. Second, most detected variants are enriched in noncoding regions, suggesting that further studies must illuminate the regulatory network. Third, applying it to embryo selection to decrease the risk of PTB could lead to an increased risk of other diseases [80]. Therefore, there is a gap between the GWAS discoveries and clinical practice. Another limitation of this study is the lack of high-quality sequencing data. We are interested in obtaining new high-quality sequencing data and providing updated results for comparative analysis in our future work.

## Conclusions

This study benchmarked the ability of ulcWGS to be used for genotype imputation. As the first study using a large cohort of human embryo samples with ulcWGS, we demonstrate its power and effectiveness in GWAS. We detected 40 significant SNPs and 11 genomic risk loci that contain independent significant SNPs and are associated with gestational age. From 166 genes mapped to the risk loci. We establish interrelationships between the mapped genes and maternal or infant diseases and provide insights into understanding the genetic associations of gestational ages. Our findings should expand current GWAS related to gestational duration and preterm trait by including Chinese samples and would therefore be helpful to future research.

## Supplementary Information

**Additional file 1: Table S1.** Genotype imputation performance at different ultra-low coverages and sample sizes. **Table S2.** WGA methods and sequencing platforms used in the PGT experiments. **Table S3.** The summary of 40 significant SNPs satisfying Bonferroni-corrected significant level of 4.526e-8. **Table S4.** The list of candidate SNPs. **Table S5.** Nonsynonymous candidate SNPs. **Table S6.** The reported genomic risk loci from GWAS catalog that were detected in our dataset. **Table S7.** A list of 166 mapped genes by positional, eQTL, and chromatin interaction mappings. **Table S8.** Data sources of genome-wide mRNA expression in Preterm birth, infant disease and breast cancer. **Table S9.** A list of the 166 mapped genes that were also identified by differentially expressed genes (DEGs) derived from analyzing the genome-wide gene expression datasets listed.

**Additional file 2: Figure S1.** Sequencing coverage statistics of the 17,844 embryos. **Figure S2.** Visualizing the linear regression model for genotype accuracy prediction. **Figure S3.** Quality control of SNV calling. **Figure S4**. Principal component analysis of all 17,844 embryo samples. **Figure S5.** Distribution of gestational age in the 1,744 born embryo samples. **Figure S6.** Quantile-Quantile plot of the 1,107,198 studied SNPs. **Figure S7.** Pie charts of the candidate SNPs. **Figure S8.** Zoom in locus plots of the 11 genomic risk loci. **Figure S9.** Circos plots of chromatin interactions and eQTL mapping. **Figure S10.** Gene set analysis of the 166 mapped genes. **Figure 11.** Co-expression network of PTB-related genes in maternal and infant subtypes.

**Additional file 3: Supplemental Methods**.

## Data Availability

The sequencing data supporting the current study have not been deposited in a public repository because of restrictions in the patient consent. The significant SNPs and candidate SNPs are listed in Additional file 1: Tables S3 and S4, respectively. The genome-wide mRNA expression dataset in preterm birth, infant disease, and breast cancer can be accessed at Gene Expression Omnibus (GEO): https://www.ncbi.nlm.nih.gov/geo/ (The dataset descriptions and accession numbers are listed in Additional file 1: Table S8).

## Abbreviations

WGS: Whole genome sequencing
NGS: Next-generation sequencing
GWAS: Genome-wide association study
bp: Base pair
SNV: Single-nucleotide variant
SNP: Single-nucleotide polymorphism
PTB: Preterm birth
PGT: Preimplantation genetic testing
MAF: Minor allele frequency
LD: Linkage disequilibrium
HWE: Hardy-Weinberg equilibrium
WGA: Whole genome amplification
PCA: Principal component analysis
eQTL: Expression quantitative trait locus
FDR: False discovery rate
GO: Gene ontology
BPD: Bronchopulmonary dysplasia
ER: Estrogen receptor
PR: Progesterone receptor
HER2: Human epidermal growth factor receptor 2
TNBC: Triple-negative breast cancer
DEG: Differentially expressed gene
AF: Allele frequency
GIAB: Genome in a Bottle
BWA: Burrows-Wheeler Aligner
GEO: Gene Expression Omnibus
NCBI: National Center for Biotechnology Information
